# A25 CRITICAL APPRAISAL OF GI ENDOSCOPY CLINICAL PRACTICE GUIDELINES DURING THE COVID-19 PANDEMIC

**DOI:** 10.1093/jcag/gwab049.024

**Published:** 2022-02-21

**Authors:** D Tham, N Gimpaya, R Gholami, C Pattni, S Seleq, R Bansal, M A Fujiyoshi, A Ramkissoon, J Lisondra, J Ariaratnam, M Scaffidi, R Khan, S Grover

**Affiliations:** St. Michael’s Hospital, Toronto, ON, Canada

## Abstract

**Background:**

Clinical Practice Guidelines (CPGs) are integral during a pandemic, offering guidance to clinicians through uncertainty. Existing literature has established that the need for rapid publication of CPGs during previous infectious disease outbreaks resulted in less rigorous guidelines. CPGs were rapidly developed since the onset of the pandemic in December 2019, providing guidance in gastrointestinal (GI) endoscopy, an area where COVID-19 may pose risk of transmission.

**Aims:**

To evaluate the quality of GI endoscopy guidelines developed during the COVID-19 pandemic and to compare these with (a) endoscopy CPGs developed prior to the pandemic; (b) CPGs for other endoscopic topics unrelated to COVID-19; and, (c) non-endoscopic CPGs published during the pandemic.

**Methods:**

We systematically searched Medline, Embase and Scopus for CPGs published by GI societies from January 1, 2018 to December 31, 2020. A grey literature search was conducted. Two authors screened full-texts. In this interim analysis, CPGs were grouped based on publication year: before 2020, or 2020. Endoscopy CPGs published in 2020 were categorized as COVID or non-COVID related. Two authors independently assessed the CPGs using the AGREE II tool, consisting of six domains for evaluating guidelines. A domain score of 60 was set as a threshold to indicate good quality.

**Results:**

There were 70 endoscopy guidelines and 27 CPGs focused on other GI topics. The mean overall scores were 69% (±12%) for endoscopy CPGs published before 2020 (n=28), and 51% (±23%) for CPGs published in 2020 (n=42). For individual AGREE II domains, mean scores for pre-2020 CPGs ranged from 33.11 (±17.39) in *Applicability* to 81.55 (±10.37) in *Clarity of Presentation*. For CPGs published during COVID-19, mean domain scores ranged from 34.18 (±10.52) in *Applicability* to 75.26 (±13.85) in *Clarity of Presentation*. 21 of 42 CPGs published in 2020 were related to COVID. Mean overall scores were 35% (±20%) for COVID-related CPGs and 67% (±13%) for non-COVID-19 CPGs. For COVID-19 CPGs, scores ranged from 27.88 (±20.31) in *Rigour of Development* to 69.58 (±10.81) in *Scope and Purpose*. For non-COVID CPGs, the scores ranged from 37.30 (±8.93) in *Applicability* to 84.52 (±5.93) in *Clarity of Presentation*.

**Conclusions:**

The difference in overall scores between COVID-19 endoscopy CPGs and non-COVID endoscopy CPGs may suggest that the urgency to disseminate COVID-19 information decreased CPG quality or completeness of reporting. This interim analysis is limited by the lack of distinction between peer-reviewed CPGs and non-peer reviewed recommendations. Given the importance of CPGs in clinical decision making, it is important to ensure that the rapid development of guidelines does not compromise quality and rigour.

**Funding Agencies:**

None

